# Supplementary material for: Efficacy and safety of tegoprazan in the treatment of gastroesophageal reflux disease: A protocol for meta-analysis and systematic review
Source: PLoS One. 2024 May 2;19(5):e0302450. doi: 10.1371/journal.pone.0302450 (PMC11065240; doi:10.1371/journal.pone.0302450)
Supplement: S1 File — (PDF) [file pone.0302450.s002.pdf]

National Institute for Health Research

University of York
Centre for Reviews and Dissemination

Systematic review

A list of fields that can be edited in an update can be found here

1. * Review title.

Give the title of the review in English

Efficacy and safety of tegoprazan in the treatment of gastroesophageal reflux pharyngitis: A protocol for meta-analysis and systematic review

2. Original language title.

For reviews in languages other than English, give the title in the original language. This will be displayed with the English language title.

3. * Anticipated or actual start date.

Give the date the systematic review started or is expected to start.

20/03/2023

4. * Anticipated completion date.

Give the date by which the review is expected to be completed.

01/06/2023

5. * Stage of review at time of this submission.

This field uses answers to initial screening questions. It cannot be edited until after registration.

Tick the boxes to show which review tasks have been started and which have been completed.

Update this field each time any amendments are made to a published record.

The review has not yet started: Yes

Review stage	Started	Completed
Preliminary searches	No	No
Piloting of the study selection process	No	No
Formal screening of search results against eligibility criteria	No	No
Data extraction	No	No
Risk of bias (quality) assessment	No	No
Data analysis	No	No

Provide any other relevant information about the stage of the review here.

6. * Named contact.

The named contact is the guarantor for the accuracy of the information in the register record. This may be any member of the review team.

HanXue Zheng

Email salutation (e.g. "Dr Smith" or "Joanne") for correspondence:

Miss

7. * Named contact email.

Give the electronic email address of the named contact.

812487914@qq.com

8. Named contact address

Give the full institutional/organisational postal address for the named contact.

Department of ent, Chongzhou People's Hospital

9. Named contact phone number.

Give the telephone number for the named contact, including international dialling code.

18780237154

10. * Organisational affiliation of the review.

Full title of the organisational affiliations for this review and website address if available. This field may be

completed as 'None' if the review is not affiliated to any organisation.

Department of ent, Chongzhou People's Hospital

Organisation web address:

11. * Review team members and their organisational affiliations.

Give the personal details and the organisational affiliations of each member of the review team. Affiliation refers to groups or organisations to which review team members belong. **NOTE: email and country now MUST be entered for each person, unless you are amending a published record.**

Miss Hanxue zheng. Department of ent, Chongzhou People's Hospital
Mr Shunqi Yuan. Department of Otorhinolaryngology, Head and Neck Surgery, Longquanyi Hospital, West China Hospital of Sichuan University
Mr Jianmin Liu. Department of Otolaryngology, Head and Neck Surgery, Deyang City People's Hospital

12. * Funding sources/sponsors.

Details of the individuals, organizations, groups, companies or other legal entities who have funded or sponsored the review.

None

Grant number(s)

State the funder, grant or award number and the date of award

13. * Conflicts of interest.

List actual or perceived conflicts of interest (financial or academic).

None

14. Collaborators.

Give the name and affiliation of any individuals or organisations who are working on the review but who are not listed as review team members. **NOTE: email and country must be completed for each person, unless you are amending a published record.**

15. * Review question.

State the review question(s) clearly and precisely. It may be appropriate to break very broad questions down into a series of related more specific questions. Questions may be framed or refined using PI(E)COS or similar where relevant.

A meta-analysis was conducted to compare the efficacy and safety of tegoprazan and placebo in the treatment of gastroesophageal reflux pharyngitis.

16. * Searches.

State the sources that will be searched (e.g. Medline). Give the search dates, and any restrictions (e.g. language or publication date). Do NOT enter the full search strategy (it may be provided as a link or attachment below.)

We will search PubMed, Embase, Cochrane Library, Web of Science and other databases until April 30, 2023. There are no restrictions on language or publication status.

17. URL to search strategy.

Upload a file with your search strategy, or an example of a search strategy for a specific database, (including the keywords) in pdf or word format. In doing so you are consenting to the file being made publicly accessible. Or provide a URL or link to the strategy. Do NOT provide links to your search **results**.

Alternatively, upload your search strategy to CRD in pdf format. Please note that by doing so you are consenting to the file being made publicly accessible.

Do not make this file publicly available until the review is complete

18. * Condition or domain being studied.

Give a short description of the disease, condition or healthcare domain being studied in your systematic review.

Reflux esophagitis is an inflammatory lesion of the esophagus caused by the reverse flow of stomach and duodenal contents into the esophagus. Under endoscopy, it is manifested as the damage of the esophageal mucosa, namely, esophageal erosion and/or esophageal ulcer. Reflux esophagitis can occur at any age, and the adult incidence increases with age. The incidence is high in Western countries and low in Asia. This regional variation may be related to both genetic and environmental factors. But in the last two decades, the global incidence has been on the rise. The elderly, obesity, smoking, drinking and mental stress are the high incidence of reflux esophagitis.

19. * Participants/population.

Specify the participants or populations being studied in the review. The preferred format includes details of both inclusion and exclusion criteria.

Adults with symptoms and signs that meet the diagnostic criteria for gastroesophageal reflux esophagitis.

20. * Intervention(s), exposure(s).

Give full and clear descriptions or definitions of the interventions or the exposures to be reviewed. The preferred format includes details of both inclusion and exclusion criteria.

Tegorazan is a new oral potassium ion competitive acid blocker, which was approved and marketed by the Korean Ministry of Food and Drug Safety in July 2018 for the treatment of gastroesophageal reflux disease and erosive esophagitis, and obtained the acceptance notice of Class 1 new drug marketing in China in January 2021. Many studies have been conducted on the clinical application of Tegolazone, and the results

show that the efficacy of Tegolazone in the treatment of non-erosive reflux disease is significantly better than placebo, the efficacy of Tegolazone in the treatment of erosive esophagitis is no worse than esomeprazole, and the efficacy of Tegolazone in the treatment of gastric ulcer is no worse than lansoprazole. The common adverse events of Tigorazone were gastrointestinal reactions and headache, but they were mild, well tolerated by patients, and had a good clinical application prospect.

21. * Comparator(s)/control.

Where relevant, give details of the alternatives against which the intervention/exposure will be compared (e.g. another intervention or a non-exposed control group). The preferred format includes details of both inclusion and exclusion criteria.

placebo

22. * Types of study to be included.

Give details of the study designs (e.g. RCT) that are eligible for inclusion in the review. The preferred format includes both inclusion and exclusion criteria. If there are no restrictions on the types of study, this should be stated.

We're going to include randomized controlled studies to compare.

23. Context.

Give summary details of the setting or other relevant characteristics, which help define the inclusion or exclusion criteria.

24. * Main outcome(s).

Give the pre-specified main (most important) outcomes of the review, including details of how the outcome is defined and measured and when these measurement are made, if these are part of the review inclusion criteria.

Complete resolution rates of major symptoms (both heartburn and regurgitation); Complete resolution rates of heartbur

Measures of effect

Please specify the effect measure(s) for you main outcome(s) e.g. relative risks, odds ratios, risk difference, and/or 'number needed to treat.

25. * Additional outcome(s).

List the pre-specified additional outcomes of the review, with a similar level of detail to that required for main outcomes. Where there are no additional outcomes please state ‘None’ or ‘Not applicable’ as appropriate to the review

adverse events

Measures of effect

Please specify the effect measure(s) for you additional outcome(s) e.g. relative risks, odds ratios, risk difference, and/or 'number needed to treat.

26. * Data extraction (selection and coding).

Describe how studies will be selected for inclusion. State what data will be extracted or obtained. State how this will be done and recorded.

Two independent evaluators independently screened literature for data extraction. By reading the title and abstract of literature, as well as the full text of literature, literature screening was conducted directly for literatures that were easy to judge. For the literature that can be included with objections, consult the relevant teachers' opinions, and screen by directly downloading and reading the full text. During the screening process, strictly follow the inclusion and exclusion criteria, extract the observation indicators of the two groups of studies, and cross-check the extracted data to ensure the consistency of the extracted data. The main contents of data extraction included: first author name, year of publication, country, Condition, sample size, follow-up, intervention measures.

27. * Risk of bias (quality) assessment.

State which characteristics of the studies will be assessed and/or any formal risk of bias/quality assessment tools that will be used.

The quality of the included studies was assessed independently by two researchers. The quality of included studies was evaluated using the bias analysis tool provided by the Cochrane Handbook for Systematic Reviews of Interventions5.1.0.(18). The evaluation included seven aspects: random sequence generation (selectivity bias), assignment concealment (selectivity bias), implementor and participant blinding (implementation bias), outcome evaluator blinding (observation bias), data results integrity (follow-up bias), selective reporting of study results (reporting bias), and other sources of bias. The seven projects were evaluated one by one according to the above criteria, so as to complete the quality evaluation of the included studies, and make the methodological quality evaluation table, bias risk chart and bias risk summary chart.

28. * Strategy for data synthesis.

Describe the methods you plan to use to synthesise data. This **must not be generic text** but should be **specific to your review** and describe how the proposed approach will be applied to your data. If meta-analysis is planned, describe the models to be used, methods to explore statistical heterogeneity, and software package to be used.

Meta-analysis was performed using RevMan 5.4 software. ?^{2} test was used to determine whether there was heterogeneity among the results. If P?0.1 and I^2 50%, multiple similar studies could be considered homogeneous, and fixed effects model was used for meta-analysis. If P?0.1 and I^2?50%, indicating clinical homogeneity, random-effects model was selected and subgroup analysis (specific interventions) was performed to find the source of heterogeneity. For continuous data, if the results were obtained by the same measurement tools, the difference in mean (MD) was used as the statistic of effect analysis. If different

measurement tools were used for the same variable, standardized mean difference (SMD) was used as the statistic of effect analysis. All effect sizes were expressed as 95% availability interval (95%CI). The Odds Ratio (OR) was used as the statistical effect size. Both data were represented by 95%CI, and P0.05 was considered statistically significant. The inverted funnel plot is used to evaluate the publication deviation. If the two sides of the inverted funnel plot are completely symmetric, the possibility of publication deviation is small; otherwise, the possibility of publication deviation is large. The stability of the research results was investigated by further sensitivity analysis.

29. * Analysis of subgroups or subsets.

State any planned investigation of 'subgroups'. Be clear and specific about which type of study or participant will be included in each group or covariate investigated. State the planned analytic approach.

If the included study indicators meet the requirements of subgroup analysis, we will conduct subgroup analysis according to different countries, sample size, country, dose of intervention measures, duration of medication, and follow-up time.

30. * Type and method of review.

Select the type of review, review method and health area from the lists below.

Type of review

Cost effectiveness

No

Diagnostic

No

Epidemiologic

No

Individual patient data (IPD) meta-analysis

No

Intervention

No

Living systematic review

No

Meta-analysis

Yes

Methodology

No

Narrative synthesis

No

Network meta-analysis

No

Pre-clinical

No

Prevention

No

Prognostic

No

Prospective meta-analysis (PMA)

No

Review of reviews

No

Service delivery

No

Synthesis of qualitative studies

No

Systematic review

Yes

Other

No

Health area of the review

Alcohol/substance misuse/abuse

No

Blood and immune system

No

Cancer

No

Cardiovascular

No

Care of the elderly

No

Child health

No

Complementary therapies

No

COVID-19

No

Crime and justice

No

Dental

No

Digestive system

No

Ear, nose and throat

Yes

Education

No

Endocrine and metabolic disorders

No

Eye disorders

No

General interest

No

Genetics

No

Health inequalities/health equity

No

Infections and infestations

No

International development

No

Mental health and behavioural conditions

No

Musculoskeletal

No

Neurological

No

Nursing

No

Obstetrics and gynaecology

No

Oral health

No

Palliative care
No

Perioperative care
No

Physiotherapy
No

Pregnancy and childbirth
No

Public health (including social determinants of health)
No

Rehabilitation
No

Respiratory disorders
No

Service delivery
No

Skin disorders
No

Social care
No

Surgery
No

Tropical Medicine
No

Urological
No

Wounds, injuries and accidents
No

Violence and abuse
No

31. Language.

Select each language individually to add it to the list below, use the bin icon to remove any added in error.
English

There is not an English language summary

32. * Country.

Select the country in which the review is being carried out. For multi-national collaborations select all the countries involved.

China

33. Other registration details.

Name any other organisation where the systematic review title or protocol is registered (e.g. Campbell, or The Joanna Briggs Institute) together with any unique identification number assigned by them. If extracted data will be stored and made available through a repository such as the Systematic Review Data Repository (SRDR), details and a link should be included here. If none, leave blank.

34. Reference and/or URL for published protocol.

If the protocol for this review is published provide details (authors, title and journal details, preferably in Vancouver format)

Add web link to the published protocol.

Or, upload your published protocol here in pdf format. Note that the upload will be publicly accessible.

No I do not make this file publicly available until the review is complete

Please note that the information required in the PROSPERO registration form must be completed in full even if access to a protocol is given.

35. Dissemination plans.

Do you intend to publish the review on completion?

No

Give brief details of plans for communicating review findings.?

36. Keywords.

Give words or phrases that best describe the review. Separate keywords with a semicolon or new line. Keywords help PROSPERO users find your review (keywords do not appear in the public record but are included in searches). Be as specific and precise as possible. Avoid acronyms and abbreviations unless these are in wide use.

37. Details of any existing review of the same topic by the same authors.

If you are registering an update of an existing review give details of the earlier versions and include a full

bibliographic reference, if available.

38. * Current review status.

Update review status when the review is completed and when it is published.New registrations must be ongoing so this field is not editable for initial submission.

Please provide anticipated publication date

Review_Ongoing

39. Any additional information.

Provide any other information relevant to the registration of this review.

40. Details of final report/publication(s) or preprints if available.

Leave empty until publication details are available OR you have a link to a preprint (NOTE: this field is not editable for initial submission). List authors, title and journal details preferably in Vancouver format.

Give the link to the published review or preprint.
