# Supplementary material for: Efficacy and safety of tegoprazan in the treatment of gastroesophageal reflux disease: A protocol for meta-analysis and systematic review
Source: PLoS One. 2024 May 2;19(5):e0302450. doi: 10.1371/journal.pone.0302450 (PMC11065240; doi:10.1371/journal.pone.0302450)
Supplement: S2 File — (DOCX) [file pone.0302450.s003.docx]

| PubMed | | |
| --- | --- | --- |
| 1 | "tegoprazan" [Supplementary Concept] | 28 |
| 2 | tegoprazan[Title/Abstract] | 42 |
| 3 | #1 OR #2 | 44 |
| 4 | "Gastroesophageal Reflux"[Mesh] | 29,055 |
| 5 | ((((((((((((((((((Gastroesophageal Reflux[Title/Abstract]) OR (Gastric Acid Reflux[Title/Abstract])) OR (Acid Reflux, Gastric[Title/Abstract])) OR (Reflux, Gastric Acid[Title/Abstract])) OR (Gastric Acid Reflux Disease[Title/Abstract])) OR (Gastro-Esophageal Reflux Disease[Title/Abstract])) OR (Gastro Esophageal Reflux Disease[Title/Abstract])) OR (Gastro-Esophageal Reflux Diseases[Title/Abstract])) OR (Reflux Disease, Gastro-Esophageal[Title/Abstract])) OR (Gastro-oesophageal Reflux[Title/Abstract])) OR (Gastro oesophageal Reflux[Title/Abstract])) OR (Reflux, Gastro-oesophageal[Title/Abstract])) OR (Gastroesophageal Reflux Disease[Title/Abstract])) OR (GERD[Title/Abstract])) OR (Reflux, Gastroesophageal[Title/Abstract])) OR (Esophageal Reflux[Title/Abstract])) OR (Gastro-Esophageal Reflux[Title/Abstract])) OR (Gastro Esophageal Reflux[Title/Abstract])) OR (Reflux, Gastro-Esophageal[Title/Abstract]) | 29,380 |
| 6 | #4 OR #5 | 39,482 |
| 7 | #3 AND #6 | 14 |

Embase

| No. | Query | Results |
| --- | --- | --- |
| #25 | #3 AND #24 | 36 |
| #24 | #4 OR #5 OR #6 OR #7 OR #8 OR #9 OR #10 OR #11 OR #12 OR #13 OR #14 OR #15 OR #16 OR #17 OR #18 OR #19 OR #20 OR #21 OR #22 OR #23 | 82535 |
| #23 | 'reflux, gastro-esophageal':ab,ti | 2 |
| #22 | 'gastro esophageal reflux':ab,ti | 3524 |
| #21 | 'gastro-esophageal reflux':ab,ti | 3528 |
| #20 | 'esophageal reflux':ab,ti | 4668 |
| #19 | 'reflux, gastroesophageal':ab,ti | 41 |
| #18 | 'gerd':ab,ti | 20839 |
| #17 | 'gastroesophageal reflux disease':ab,ti | 20038 |
| #16 | 'reflux, gastro-oesophageal':ab,ti | 7 |
| #15 | 'gastro oesophageal reflux':ab,ti | 5754 |
| #14 | 'gastro-oesophageal reflux':ab,ti | 5761 |
| #13 | 'reflux disease, gastro-esophageal':ab,ti | 1 |
| #12 | 'gastro-esophageal reflux diseases':ab,ti | 21 |
| #11 | 'gastro esophageal reflux disease':ab,ti | 1927 |
| #10 | 'gastro-esophageal reflux disease':ab,ti | 1929 |
| #9 | 'gastric acid reflux disease':ab,ti | 1 |
| #8 | 'reflux, gastric acid':ab,ti | 8 |
| #7 | 'acid reflux, gastric':ab,ti | 4 |
| #6 | 'gastric acid reflux':ab,ti | 82 |
| #5 | 'gastroesophageal reflux':ab,ti | 31909 |
| #4 | 'gastroesophageal reflux'/exp | 75574 |
| #3 | #1 OR #2 | 74 |
| #2 | 'tegoprazan':ab,ti | 55 |
| #1 | 'tegoprazan'/exp | 72 |

Cochrane library

D Search Hits

#1 (tegoprazan):ti,ab,kw 64

#2 MeSH descriptor: [Gastroesophageal Reflux] explode all trees 2555

#3 (Gastroesophageal Reflux):ti,ab,kw OR (Gastric Acid Reflux):ti,ab,kw OR (Acid Reflux, Gastric):ti,ab,kw OR (Reflux, Gastric Acid):ti,ab,kw OR (Gastric Acid Reflux Disease):ti,ab,kw 5049

#4 (Gastro-Esophageal Reflux Disease):ti,ab,kw OR (Gastro Esophageal Reflux Disease):ti,ab,kw OR (Gastro-Esophageal Reflux Diseases):ti,ab,kw OR (Reflux Disease, Gastro-Esophageal):ti,ab,kw OR (Gastro-oesophageal Reflux):ti,ab,kw 1167

#5 (Gastro oesophageal Reflux):ti,ab,kw OR (Reflux, Gastro-oesophageal):ti,ab,kw OR (Gastroesophageal Reflux Disease):ti,ab,kw OR (GERD):ti,ab,kw OR (Reflux, Gastroesophageal):ti,ab,kw 5349

#6 (Esophageal Reflux):ti,ab,kw OR (Gastro-Esophageal Reflux):ti,ab,kw OR (Gastro Esophageal Reflux):ti,ab,kw OR (Reflux, Gastro-Esophageal):ti,ab,kw 1861

#7 #2 or #3 or #4 or #5 or #6 5873

#8 #1 and #7 18

Web of science

| NO | Query | Result |
| --- | --- | --- |
| #1 | TS=(tegoprazan ) | 46 |
| #2 | TS=(Gastroesophageal Reflux) OR TS=(Gastric Acid Reflux) OR TS=(Acid Reflux, Gastric) OR TS=(Reflux, Gastric Acid) OR TS=(Gastric Acid Reflux Disease) OR TS=(Gastro-Esophageal Reflux Disease) OR TS=(Gastro Esophageal Reflux Disease) OR TS=(Gastro-Esophageal Reflux Diseases) OR TS=(Reflux Disease, Gastro-Esophageal) OR TS=(Gastro-oesophageal Reflux) OR TS=(Gastro oesophageal Reflux) OR TS=(Reflux, Gastro-oesophageal) OR TS=(Gastroesophageal Reflux Disease) OR TS=(GERD) OR TS=(Reflux, Gastroesophageal) OR TS=(Esophageal Reflux) OR TS=(Gastro-Esophageal Reflux) OR TS=(Gastro Esophageal Reflux) OR TS=(Reflux, Gastro-Esophageal) | 18653 |
| #3 | #1 AND #2 | 16 |
